# TRPV4 is a regulator in *P. gingivalis* lipopolysaccharide‐induced exacerbation of macrophage foam cell formation

**DOI:** 10.14814/phy2.14069

**Published:** 2019-04-13

**Authors:** Nabyendu Gupta, Rishov Goswami, Mazen O. Alharbi, Debabrata Biswas, Shaik O. Rahaman

**Affiliations:** ^1^ Department of Nutrition and Food Science University of Maryland College Park Maryland; ^2^ Department of Animal and Avian Sciences University of Maryland College Park Maryland

**Keywords:** Calcium channel, foam cells, lipopolysaccharide, matrix stiffness, TRPV4

## Abstract

*Porphyromonas gingivalis* (P.g), a major causative agent of periodontitis, has been linked to atherosclerosis, a chronic inflammatory vascular disease. Recent studies have suggested a link between periodontitis and arterial stiffness, a risk factor for atherosclerosis. However, the mechanisms by which P.g infection contributes to atherogenesis remain elusive. The formation of lipid‐laden macrophage “foam cells” is critically important to development and progression of atherosclerosis. We have obtained evidence that TRPV4 (transient receptor potential channel of the vanilloid subfamily 4), a mechanosensitive channel, is a regulator of macrophage foam cell formation both in response to P.g‐derived lipopolysaccharide (PgLPS) or to an increase in matrix stiffness. Importantly, we found that TRPV4 activity (Ca^2+^ influx) was increased in response to PgLPS. Genetic deletion or chemical antagonism of TRPV4 channels blocked PgLPS‐triggered exacerbation of oxidized LDL (oxLDL)‐mediated foam cell formation. Mechanistically, we found that (1) TRPV4 regulated oxLDL uptake but not its cell surface binding in macrophages; (2) reduced foam cell formation in TRPV4 null cells was independent of expression of CD36, a predominant receptor for oxLDL, and (3) co‐localization of TRPV4 and CD36 on the macrophage plasma membrane was sensitive to the increased level of matrix stiffness occurring in the presence of PgLPS. Altogether, our results suggest that TRPV4 channels play an essential role in P.g‐induced exacerbation of macrophage foam cell generation through a mechanism that modulates uptake of oxLDL.

## Introduction

Atherosclerosis, a chronic inflammatory vascular disease, accounts for the majority of deaths linked to cardiovascular disease (CVD) (Lusis 2000; Moore and Tabas 2011; Falk et al. 2013). Tissue macrophages recognize and take up oxidized low‐density lipoproteins (oxLDL) through various scavenger receptors (SR) such as CD36 and SR‐A, and contribute to generation of lipid‐loaded “foam cells,” a critical early event in the development of artherosclerotic lesions (Lusis 2000; Collot‐Teixeira et al. 2007; McLaren et al. 2011; Moore and Tabas 2011; Falk et al. 2013). Progressive generation and buildup of macrophage foam cells along with other inflammatory changes such as generation of cellular debris, lipids, expression of inflammatory cytokines, and deposition of calcium in the aortic intimal areas initiate the formation of atherosclerotic plaques, and consequently cause development of atherosclerosis and related pathologies (Lusis 2000; Bobryshev 2006; Libby 2008; Moore and Tabas 2011; Moore et al. 2013).

Approximately 50% of CVD patients lack traditional risk factors (Lusis 2000; McLaren et al. 2011; Moore and Tabas 2011; Moore et al. 2013; Ruparelia et al. 2017; Thomas and Lip 2017). Numerous clinical and experimental studies have shown that infection with various bacterial pathogens including *Porphyromonas gingivalis* may serve as an additional risk factor in the development and progression of atherosclerosis (Tonetti 2009; Kebschull et al. 2010; Hayashi et al. 2011, 2012; Fukasawa et al. 2012; Teeuw et al. 2014; Chukkapalli et al. 2015; Hajishengallis 2015; Schmitt et al. 2015; Houcken et al. 2016). Emerging experimental and epidemiological studies suggest an association between periodontitis, a chronic infection of the periodontium, and atherosclerosis, even after controlling for traditional CVD‐related risk factors (Tonetti 2009; Kebschull et al. 2010; Hayashi et al. 2011, 2012; Fukasawa et al. 2012; Teeuw et al. 2014; Walters and Lai 2015). *P. gingivalis,* a predominant causative factor of periodontitis, has been reported to accelerate atherosclerosis in animal models (Hayashi et al. 2011, 2012; Fukasawa et al. 2012; Chukkapalli et al. 2015). However, the precise mechanism whereby *P. gingivalis* induces atherosclerosis is not well understood. Recent epidemiologic studies suggest a link between periodontal disease and the development of stiffness in arterial tissues (Schmitt et al. 2015; Houcken et al. 2016). Studies also suggest that arterial stiffness is an underappreciated risk factor for various cardiovascular diseases including atherosclerosis (Kothapalli et al. 2012; Hansen and Taylor 2016; Palombo and Kozakova 2016; Tedla et al. 2017). Interestingly, macrophages, a critical cell type in the development of atherosclerosis, have been shown to directly respond to changes in their internal and external biomechanical environment (Doherty et al. 1994; Blakney et al. 2012; Pi et al. 2014; Hind et al. 2015; Previtera and Sengupta 2015; Adlerz et al. 2016; Scheraga et al. 2016). Published reports by our group and others have shown that numerous proatherogenic macrophage functions including migration, phagocytosis, and proliferation are influenced by matrix stiffness, implying that stiffness may play a critical role in determining the proatherogenic response of macrophages in the context of periodontitis/atherosclerosis (Doherty et al. 1994; Blakney et al. 2012; Pi et al. 2014; Hind et al. 2015; Previtera and Sengupta 2015; Adlerz et al. 2016; Scheraga et al. 2016). Additionally, it has been shown that bacterial LPS can increase macrophage rigidity and stiffening of vasculature in vivo (Doherty et al. 1994; Meng et al. 2015).

Various macrophage functions including migration, differentiation, apoptosis, and inflammatory responses are sensitive to Ca^2+^ signaling (Shi et al. 1996; Enomoto et al. 1999; Melendez and Tay 2008; Nunes and Demaurex 2010; Chiang et al. 2012; Scheraga et al. 2016). Calcium overload has been linked to the genesis of atherosclerotic lesions (Fleckenstein‐Grün et al. 1994; Shi et al. 1996). As well, Ca^2+^ signaling is known to regulate phagocytosis and foam cell formation (Fleckenstein‐Grün et al. 1994; Shi et al. 1996; Yang et al. 2000; Melendez and Tay 2008; Nunes and Demaurex 2010; Rahaman et al. 2011a,b). Macrophages regulate their overall calcium homeostasis through various ion channels and membrane pumps (Tang et al. 2013). TRPV4, a Ca^2+^‐permeable mechanosensitive cation channel, is ubiquitously expressed in various cell types, including macrophages (Liedtke and Friedman 2003; Liedtke et al. 2003; Suzuki et al. 2003; Köhler et al. 2006; Liedtke 2008; Auer‐Grumbach et al. 2010; Everaerts et al. 2010; Lamandé et al. 2011; Thorneloe et al. 2012; Adapala et al. 2013; Garcia‐Elias et al. 2014; Du et al. 2016; Goswami et al. 2016, 2017; Xu et al. 2016; Sharma et al. 2017). Published data from our group and others have shown that TRPV4 plays critical roles in numerous pathophysiological conditions (Liedtke and Friedman 2003; Liedtke et al. 2003; Suzuki et al. 2003; Köhler et al. 2006; Liedtke 2008; Auer‐Grumbach et al. 2010; Everaerts et al. 2010; Lamandé et al. 2011; Thorneloe et al. 2012; Adapala et al. 2013; Garcia‐Elias et al. 2014; Du et al. 2016; Goswami et al. 2016, 2017; Xu et al. 2016; Sharma et al. 2017). Impairment in TRPV4 channel function is associated with endothelial dysfunction, oxidized LDL‐induced macrophage foam cell formation, and vascular diseases (Zhang et al. 2009; Ye et al. 2012; Du et al. 2016; Goswami et al. 2017). However, although the data are suggestive, it is not known whether TRPV4 plays a role in *P. gingivalis* ‐derived lipopolysaccharide (PgLPS)‐triggered proatherogenic responses in macrophages. Here, we sought to determine whether and by what mechanism TRPV4 plays a role in PgLPS‐triggered exacerbation of oxLDL‐induced macrophage foam cell formation. As TRPV4 channels are sensitive to changes in the cellular internal and external biomechanical environment (Liedtke and Friedman 2003; Liedtke et al. 2003; Liedtke 2008; Matthews et al. 2010; Adapala et al. 2013; Rahaman et al. 2014; Goswami et al. 2017; Sharma et al. 2017), we hypothesized that PgLPS‐sensitized TRPV4 may upregulate oxLDL‐induced inflammatory and atherogenic macrophage functions. We found that this was indeed the case, as TRPV4 plays an essential role in PgLPS‐triggered exacerbation of oxLDL‐induced macrophage foam cell formation by modulating uptake of oxLDL.

## Materials and Methods

### Antibodies and reagents

TRPV4‐specific antagonist, GSK2193874 (GSK219), TRPV4‐specific agonist, GSK1016790A (GSK101), nucleus‐specific stain, DiI (1,1′‐Dioctadecyl‐3,3,3′,3′‐tetramethylindocarbocyanine perchlorate), and Ca^2+^ ionophore, A23187 (A23) were purchased from Sigma‐Aldrich (St. Louis, MO). The following antibodies were purchased: anti‐TRPV4 (Alomone Labs; Jerusalem, Israel); anti‐*β*‐Actin HRP‐conjugate (Santa Cruz Biotechnology; Dallas, TX); anti‐CD36 (BD Pharmingen); goat‐anti rabbit Alexa Flour 488 and goat‐anti Mouse Alexa Flour 594 (Life technologies). FLIPR Calcium 6 assay kit was purchased from Molecular Devices (Sunnyvale, CA) and macrophage colony‐stimulating factor (M‐CSF) was obtained from R & D. We prepared copper‐oxidized LDL (oxLDL) by incubating human native LDL (Stemcell Technologies; Vancouver, BC, Canada) with CuSO4 (5 *μ*m) for 12 h at 37°C, as described previously (Rahaman et al. 2006, 2013). Cell culture media (RPMI‐1640) and other cell culture‐related reagents were purchased from Gibco. We obtained collagen‐coated polyacrylamide hydrogels (0.5‐50 kPa) from Matrigen Life Technologies (Brea, CA). *P. gingivalis* ‐derived lipopolysaccharide was purchased from InvivoGen (San Diego, CA).

### Animal and cell culture

We acquired the TRPV4 knockout (TRPV4 KO) mouse line from Zhang (Medical College of Wisconsin, Milwaukee, WI). The original creator of these mice on a C57BL/6 background was Suzuki (Jichi Medical University, Tochigi, Japan) (Suzuki et al. 2003). Congenic wild type (WT) mice were purchased from Charles River Laboratories (Wilmington, Massachusetts, USA). All animal experiments were performed following Institutional Animal Care and Use Committee guidelines approved by the University of Maryland review committee. Murine resident macrophages (MRMs) and bone marrow‐derived macrophages (BMDMs) were isolated as we described previously (Rahaman et al. 2006, 2013). Briefly, thioglycollate‐elicited peritoneal MRMs from background‐matched control WT and TRPV4 KO mice were plated on coverslips in 12‐well plates in RPMI‐1640 medium containing 10% FCS. After 2 h of incubation, nonadherent MRMs were washed out, fresh medium was added, and incubation was continued for 24 h. For BMDM culture, femurs and femur heads from 6 to 7 week old WT and TRPV4 KO mice were collected, and bone marrow was flushed out with RPMI‐1640. The suspended bone marrow cells were filtered through a 70 *μ*m strainer. The single cell suspension was centrifuged, plated in RPMI‐1640 medium containing M‐CSF (20 ng/mL), and incubated for 7–8 days to differentiate into macrophages.

### Measurement of intracellular calcium

Calcium responses in BMDMs were recorded on a FlexStation system (Molecular Devices, Sunnyvale, CA) using the FLIPR Calcium 6 Assay Kit. Since TRPV4‐elicited Ca^2+^ influx was greater in BMDMs than MRMs, we used BMDMs for Ca^2+^ influx studies. Cells were seeded into collagen coated (10 *μ*g/mL) 96‐well plastic plates with RPMI containing 10% serum and 25 ng/mL M‐CSF. BMDMs required 7–8 days for complete differentiation and adherence to the surface. After cellular adhesion the medium was replaced with 0.5% serum containing RPMI and 500 ng/mL pgLPS to select wells. Adhered BMDMs were incubated for 90 min with FLIPR Calcium 6 dye containing a buffer system (HBSS and 20 mmol/L HEPES) and 2.5 mmol/L probenecid. The selective TRPV4 agonist, GSK101, was used to induce cytosolic Ca^2+^ influx. To block TRPV4‐generated Ca^2+^ influx, BMDMs were pretreated with vehicle or TRPV4 antagonist, GSK219, for 45 min. Cytosolic Ca^2+^ influx was measured as relative fluorescence units (RFU), and was recorded by measuring ΔF/F (Max‐Min) as we described previously (Rahaman et al. 2014; Goswami et al. 2017; Sharma et al. 2017).

### Foam cell assays

MRMs from TRPV4 KO and WT mice were seeded either on collagen‐coated (10 *μ*g/mL) glass coverslips or on polyacrylamide gels of varying stiffness (0.5 and 8 kPa) in RPMI 1640. After the initial 48 h incubation, 50 *μ*g/mL of control native LDL (nLDL) or oxLDL with or without 500 ng/mL PgLPS were added, and incubation was continued for 20 h, as we published previously (Rahaman et al. 2006, 2013). To identify foam cells, MRMs were stained with Oil Red O following our published method (Rahaman et al. 2006, 2013).

### Binding and uptake of oxLDL

To examine binding, MRMs seeded on glass coverslips with or without 500 ng/mL pgLPS were incubated with DiI‐labeled oxLDL (DiI‐oxLDL) (5 *μ*g/mL) for 60 min at 4°C (Rahaman et al. 2006, 2013). To assess uptake, cells were treated similarly following DiI‐oxLDL treatment, but were incubated at 37°C, and were imaged at 30 min, as we previously published (Rahaman et al. 2006, 2013). For both binding and uptake assay, fluorescence intensity was examined by Zeiss Axio Observer microscope (63x), and quantified by NIH ImageJ software.

### Immunoblot (IB) analysis

BMDMs seeded on plates with or without PgLPS (0, 250, 1000 ng/mL) were incubated for 48 h. Cells were lysed in a buffer system containing 20 mmol/L Tris‐HCl (pH 7.5), 150 mmol/L NaCl, 1 mmol/L EDTA, 1 mmol/L EGTA, 1% NP‐40, 2.5 mmol/L sodium pyrophosphate, 1 mmol/L *β*‐glycerophosphate, 1 mmol/L Na_3_VO_4_, and 1 *μ*g/mL leupeptin. Whole cell lysate proteins were separated in a SDS‐polyacrylamide gel, and probed with antibodies specific to TRPV4, actin, or CD36.

### Immunofluorescence analysis

MRMs were seeded on polyacrylamide gels (0.5, 8, and 50 kPa) for 48 h, fixed with 3% paraformaldehyde, and incubated with antibodies specific to TRPV4 (1:100) or CD36 (1:100) protein. Goat‐anti rabbit Alexa Fluor 488 (1:300) or goat‐anti Mouse Alexa Flour 594 (1:300) was used as the secondary antibody. We used prolong diamond antifade reagent (Life technologies) with DAPI as the mounting reagent. We quantified immunofluorescence intensity of stained cells by ImageJ software (NIH), and the results are presented as Integrated Density (Int. Density: the product of Area and Mean Gray Value).

### Quantitative real‐time polymerase chain reaction (qRT‐PCR)

We used RNeasy Micro kit (Qiagen) to harvest total RNA from WT and TRPV4 knockout MRMs. We performed one‐step qRT‐PCR analysis using QuantiNova SYBR Green RT‐PCR Kit (Qiagen) according to the manufacturer's instructions. CD36, TRPV4, TNF‐*α*, IL‐6, IL‐1*β*, and control GAPDH primers were purchased from Thermofisher (USA), and qRT‐PCR was carried out per the manufacturer's instructions using TaqMan gene Expression Assay (Applied Biosystems). Normalized mRNA expression of CD36 or TRPV4 was determined using mRNA for GAPDH as the internal control. We used the comparative C_T_ method described in the ABI 7900 HT sequence detection system user bulletin.

### Data analysis

All data are reported as mean ± SEM. Statistical analysis between control and experimental groups was performed using the Student's *t*‐test or ANOVA using Prism software; p ≤ 0.05 was considered to indicate significance.

## Results

*P. gingivalis* lipopolysaccharide‐triggered exacerbation of oxidized LDL‐induced macrophage foam cell formation is reliant on TRPV4.

**Figure 1:**
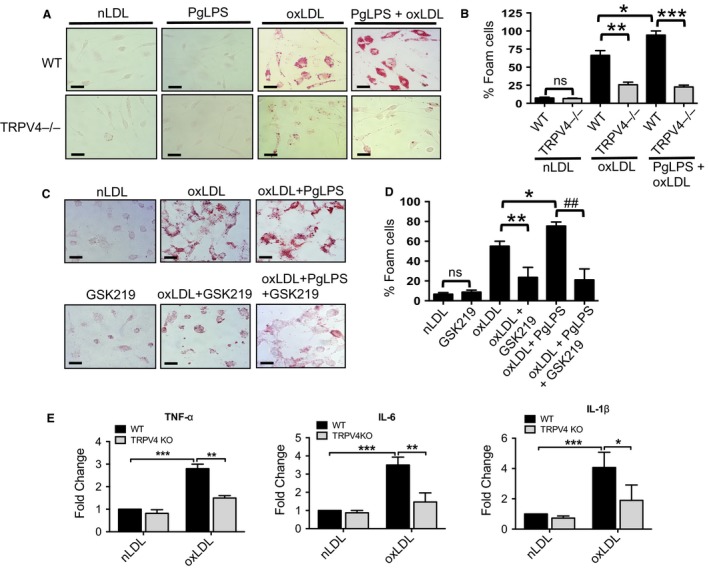
PgLPS‐triggered exacerbation of oxidized LDL‐mediated macrophage foam cell formation is dependent on TRPV4. (A) WT and TRPV4 KO (TRPV4−/−) primary resident macrophages (MRMs) were cultured for 48 h on collagen‐coated (10 *μ*g/mL) glass coverslips with or without PgLPS (500 ng/mL), and then were incubated for an additional 20 h in the presence of native low‐density lipoproteins (nLDL, 50 *μ*g/mL) or oxidized LDL (oxLDL, 50 *μ*g/mL). MRMs were stained with Oil‐Red‐O to examine macrophage foam cell formation. Representative images from five different fields per condition are shown (original magnification, 40×). (B) Bar graph shows quantitation of results in Figure 1A. Results are expressed as mean ± SEM. *P < 0.05 for oxLDL‐treated WT cells with PgLPS versus oxLDL‐treated WT cells without PgLPS, **P < 0.01 for oxLDL‐treated WT cells versus oxLDL‐treated TRPV4‐/‐ cells, and ***P < 0.001 for oxLDL treated TRPV4−/− versus WT cells in presence of PgLPS;* n* = 500 cells/condition, Student's *t*‐test. ns, not significant. (C) MRMs were cultured for 48 h with or without PgLPS as above, and were then pretreated with or without TRPV4 antagonist, GSK219 (5 *μ*mol/L) and oxLDL or nLDL (50 *μ*g/mL) for 24 h. Cells were stained with Oil‐Red‐O to examine foam cell generation. Representative images from five different fields per condition are presented (original magnification, 40×). (D) Quantitation of results from Figure 1C. *P < 0.05 for oxLDL‐treated cells with PgLPS versus oxLDL‐treated cells without PgLPS, **P < 0.01 for oxLDL‐treated cells versus oxLDL‐treated cells with GSK219, and ^##^ *P* < 0.001 for oxLDL plus PgLPS‐treated cells versus cells with GSK219; *n* = 500 cells/condition, Student's *t*‐test. (E) qRT‐PCR analysis was performed to determine levels of TNF‐*α*, IL‐6, and IL‐1*β* mRNA in WT and TRPV4 KO MRMs in the presence of nLDL or oxLDL using a TaqMan gene Expression Assay. All Ct values were normalized to gapdh levels. The experiment was repeated three times in quadruplicate. Student's *t*‐test, *P < 0.05, **P < 0.01 and ***P < 0.001.

We compared oxLDL‐induced foam cell formation in WT and TRPV4 KO (TRPV4−/−) MRMs in the presence or absence of stimulation with *P. gingivalis ‐*derived LPS (PgLPS). As expected, we found a fourfold increase in foam cell generation in oxLDL treated WT MRMs compared to control native LDL (nLDL) treated cells (Fig. 1A and B). The combined treatment with PgLPS and oxLDL further increased macrophage foam cells in WT MRMs compared to untreated controls (Fig. 1A and B). The results showed that the deficiency of TRPV4 function in TRPV4−/− MRMs abrogated (by more than twofold) macrophage foam cell formation regardless of treatment with oxLDL alone or oxLDL plus PgLPS (Fig. 1A and B). Similarly, we observed that TRPV4 antagonism by pharmacologic inhibition (GSK219 treatment) in MRMs abrogated PgLPS‐induced exacerbation of oxLDL‐mediated macrophage foam cell formation (Fig. 1C and D). We assessed expression levels of TNF‐*α*, IL‐6, and IL‐1*β* in WT and TRPV4 KO MRMs by qRT‐PCR analysis. We detected reduced expression levels of all three mRNAs in WT cells compared to TRPV4 KO treated with oxLDL for 24 h (Fig. 1E). Taken together, these findings indicate that TRPV4 plays a role in oxLDL‐induced inflammatory protein expression and in PgLPS‐induced exacerbation of oxLDL‐mediated macrophage foam cell generation.

### TRPV4 deficiency prevents *P. gingivalis* lipopolysaccharide‐induced exacerbation of foam cell formation in response to augmented matrix stiffness

**Figure 2:**
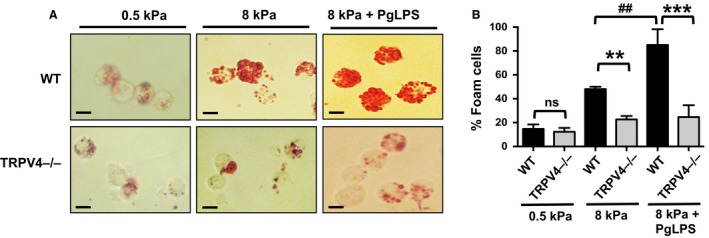
TRPV4 deficiency prevents PgLPS‐induced exacerbation of foam cell formation in response to increased matrix stiffness. (A) WT and TRPV4−/− MRMs were grown on collagen‐coated (10 *μ*g/mL) soft (0.5 kPa) and stiff (8 kPa) polyacrylamide gels for 24 h with or without PgLPS (500 ng/mL), followed by incubation with oxLDL (50 *μ*g/mL) for 24 h. Oil‐Red‐O staining was performed to examine foam cell generation. Representative images from five different fields per condition are shown (original magnification, 40×). (B) Bar graph shows quantitation of results in Figure 2A. Results are expressed as mean ± SEM. **P < 0.01 for TRPV4−/− cells on 8 kPa versus WT on 8 kPa; ***P < 0.001 for TRPV4−/− on 8 kPa with PgLPS versus WT cells on 8 kPa with PgLPS; ^##^ *P* < 0.01 for WT cells on 8 kPa versus WT cells on 8 kPa with PgLPS;* n* = 50 cells/condition, Student's *t*‐test.

We tested whether PgLPS treatment would cause enhanced macrophage foam cell formation in response to increased matrix stiffness in the presence of TRPV4, a matrix stiffness sensitive channel. In cells growing on a stiff matrix (8 kPa), we found a twofold enhancement in oxLDL‐induced foam cell formation in WT cells in response to PgLPS compared to oxLDL alone (Fig. 2A and B). The results showed that a deficiency of TRPV4 function (TRPV4−/− MRMs) abrogated (by fourfold) oxLDL‐induced macrophage foam cell formation in response to PgLPS compared to WT cells. Taken together, these results suggest that biomechanical stimuli such as matrix stiffness may modulate exacerbation of PgLPS‐induced foam cell formation in a TRPV4‐dependent manner.

### TRPV4‐dependent Ca^2+^ influx is increased in response to *P. gingivalis* lipopolysaccharide

**Figure 3:**
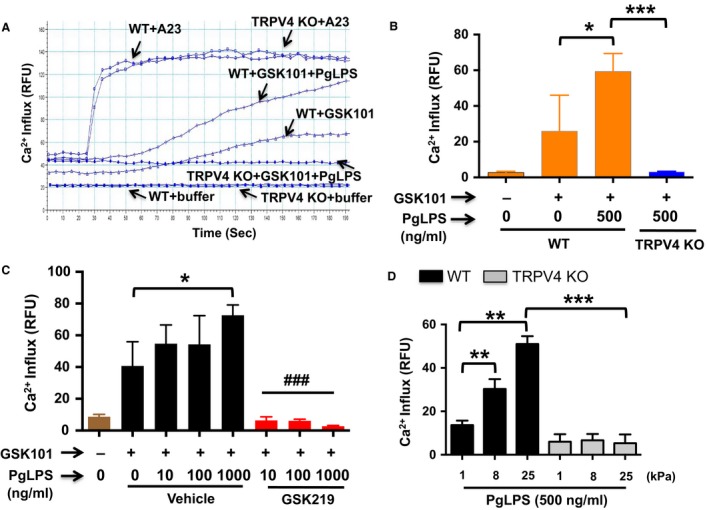
PgLPS‐triggered TRPV4‐elicited Ca^2+^ influx is increased in a matrix stiffness‐dependent manner. (A–B) FlexStation 3 recordings of Calcium 6 dye‐loaded BMDMs shows effect of TRPV4 agonist, GSK101 (10 nmol/L), on Ca^2+^ influx in WT and TRPV4−/− cells grown with or without PgLPS for 24 h. *P < 0.05 for WT cells versus WT plus PgLPS; ***P < 0.001 for TRPV4−/− with PgLPS versus WT with PgLPS. A23187 (or A23), a calcium ionophore, was used as a positive control. (C) Inhibition of PgLPS‐induced TRPV4‐elicited Ca^2+^ influx by GSK219. *P < 0.05 for WT cells versus WT plus PgLPS; ^###^ *P* < 0.001 for WT with PgLPS versus WT with PgLPS plus GSK219. (D) WT and TRPV4 KO MRMs were seeded (10,000 cells per well) on collagen‐coated (10 *μ*g/mL) polyacrylamide gels of various stiffness (1, 8, and 25 kPa). GSK101‐induced (10 nmol/L) Ca^2+^ influx was recorded as in Figure 3A. **P < 0.01 for cells grown on stiff (8 kPa and 25 kPa) hydrogels vs. soft (1 kPa), ***P < 0.001 for TRPV4 KO vs. WT cells grown on 25 kPa hydrogels. All experiments were repeated three times in quadruplicate.

We tested whether a physiological inflammatory stimulus mediated by PgLPS affected TRPV4‐dependent Ca^2+^ influx. To record the presence of TRPV4‐mediated Ca^2+^ influx in BMDMs, we detected Ca^2+^ influx induced by a selective TRPV4 channel agonist, GSK101, with or without PgLPS pretreatment (Thorneloe et al. 2012; Goswami et al. 2017). Results indicated that cytosolic Ca^2+^ influx was potentiated in the PgLPS‐treated cells compared to the unstimulated group (Fig. 3A and B). As we expected, results showed that Ca^2+^ influx was undetectable in similarly treated TRPV4 KO BMDMs (Fig. 3A and B). Furthermore, GSK101‐induced Ca^2+^ influx was inhibited by the selective TRPV4 channel antagonist, GSK219, in PgLPS pretreated BMDMs compared to antagonist‐untreated controls (Thorneloe et al. 2012; Goswami et al. 2017) (Fig. 3C). These results confirmed that loss of TRPV4 function by genetic deficiency or by pharmacologic antagonism abrogated PgLPS‐induced Ca^2+^ influx. In addition, we observed that PgLPS‐induced TRPV4‐elicited Ca^2+^ influx was augmented in a matrix stiffness‐dependent manner when macrophages were grown on stiffer matrices (8 and 25 kPa) compared to macrophages grown on soft matrix (1 kPa) (Fig. 3D). Furthermore, genetic deletion of TRPV4 in BMDMs, specifically reduced matrix stiffness‐induced Ca^2+^ influx (Fig. 3D). Altogether, these findings showed that TRPV4‐dependent Ca^2+^ influx was potentiated by both PgLPS stimulation and matrix stiffness in mouse primary macrophages.

### Reduction in macrophage foam cell formation in TRPV4 deficient cells is independent of expression of CD36

**Figure 4:**
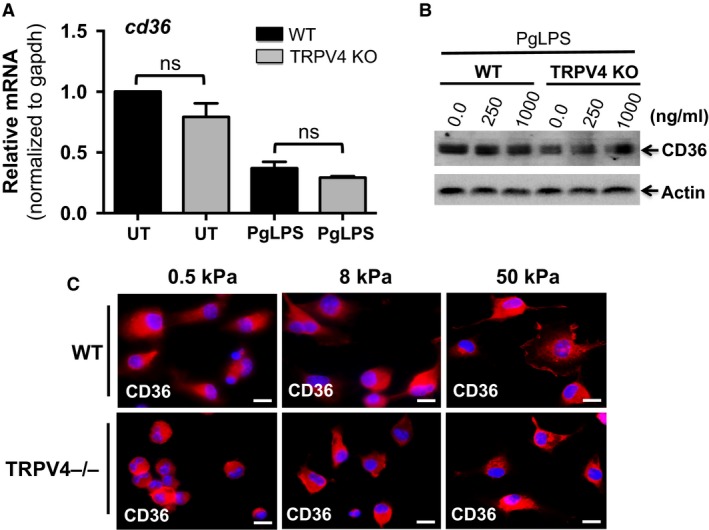
Reduced foam cell formation in TRPV4 KO cells is independent of expression of CD36. (A) qRT‐PCR analysis was performed to determine levels of CD36 in WT and TRPV4 KO MRMs using a TaqMan gene Expression Assay. All Ct values were normalized to gapdh levels. The experiment was repeated three times in quadruplicate. ns, not significant; Student's *t*‐test. (B) Representative immunoblots from three independent experiments show expression of CD36 and actin proteins in whole cell extract from WT and TRPV4 KO MRMs with or without PgLPS treatment. (C) WT and TRPV4−/− MRMs were maintained on various stiffness collagen‐coated (10 *μ*g/mL) polyacrylamide gels (0.5, 8, and 50 kPa) for 48 h, and then stained with anti‐CD36 IgG (red color). Representative immunofluorescence images from three independent experiments are shown (original magnification, 40×).

We assessed expression levels of CD36 in WT and TRPV4 KO MRMs by qRT‐PCR, immunoblot, and immunofluorescence analysis. We detected similar expression levels of CD36 mRNA and CD36 protein in both WT and TRPV4 KO macrophages treated or not with PgLPS for 24 h (Fig. 4A and B). Since we found that increasing matrix stiffness upregulated the ability of TRPV4 to augment PgLPS‐induced Ca^2+^ influx (Fig. 3D), we evaluated whether changes in matrix stiffness would cause enhanced expression levels of CD36 protein in MRMs by immunofluorescence analysis. We found that culture of both WT and TRPV4 KO macrophages on a stiffer matrix (8 or 50 kPa) compared to a softer matrix (0.5 kPa) for 24 h resulted in similar levels of CD36 protein expression (Fig. 4C). Altogether, these results indicated that reduced foam cell formation in TRPV4 deficient macrophages is independent of CD36 expression.

### Plasma membrane colocalization of TRPV4 and CD36 is sensitive to changes in matrix stiffness in PgLPS‐treated MRMs

**Figure 5:**
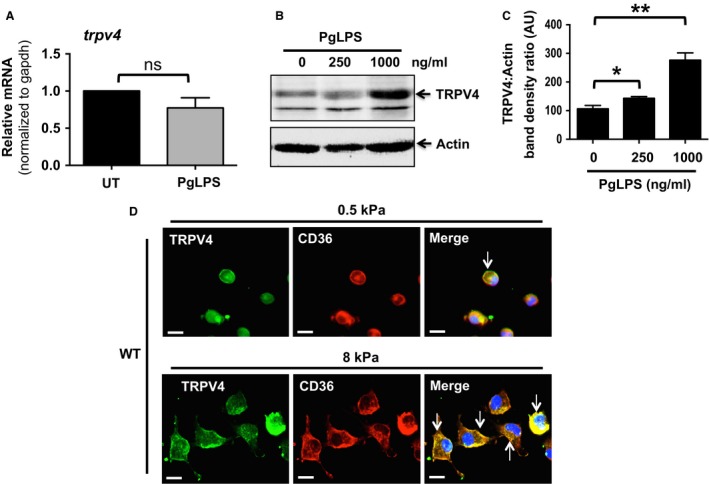
Increased plasma membrane colocalization of TRPV4 and CD36 in PgLPS‐treated MRMs in response to stiff matrix. (A) qRT‐PCR analysis was performed to determine TRPV4 mRNA levels in WT MRMs with or without PgLPS treatment. All Ct values were normalized to gapdh mRNA levels. (B) Representative immunoblots from three independent experiments show expression of TRPV4 and actin proteins in whole cell extracts from WT MRMs with or without PgLPS treatment. Actin levels were served as a loading control. (C) Bar graph shows quantitation of results in Figure 5B. Results are expressed as mean ± SEM. *P < 0.05 for cells with 250 ng/mL PgLPS versus without PgLPS; **P < 0.01 for cells with 1000 ng/mL PgLPS versus without PgLPS; Student's *t*‐test. (D) WT MRMs were maintained on various stiffness collagen‐coated (10 *μ*g/mL) polyacrylamide gels (0.5 and 8 kPa) with 1000 ng/mL PgLPS for 48 h, and then immunostained with anti‐CD36 and TRPV4 IgG. Representative immunofluorescence images from three independent experiments are shown (original magnification, 40×).

To determine whether TRPV4 plays a role in macrophage foam cell formation, we first detected the expression levels of TRPV4 with or without PgLPS stimulation in WT MRMs. Our results showed similar expression levels of TRPV4 mRNA with or without PgLPS treatment (Fig. 5A). Interestingly, immunoblot data showed that treatment with PgLPS for 24 h increased TRPV4 protein expression in a dose‐dependent manner (Fig. 5B and C). Numerous factors have been reported to modulate plasma membrane accumulation of TRPV4 and CD36 (Cuajungco et al. 2006; Ring et al. 2006; Yamada et al. 2009). We assessed changes in plasma membrane accumulation and possible colocalization of TRPV4 and CD36 in response to increasing matrix stiffness in MRMs simulated with PgLPS. Immunofluorescence data showed that exposure of MRMs to a stiff matrix (8 kPa) compared to a soft matrix (0.5 kPa) for 24 h promoted increased plasma membrane enrichment and colocalization of TRPV4 and CD36. These data suggest that changes in matrix stiffness may provide a potential mechanism for functional crosstalk between TRPV4 and CD36 (Fig. 5D). We also noticed that exposure of MRMs to a stiff matrix (8 kPa) compared to a soft matrix (0.5 kPa) for 24 h resulted in an increase in cell surface area.

### TRPV4 regulates PgLPS‐induced oxLDL internalization but not its cell surface binding in macrophages

**Figure 6:**
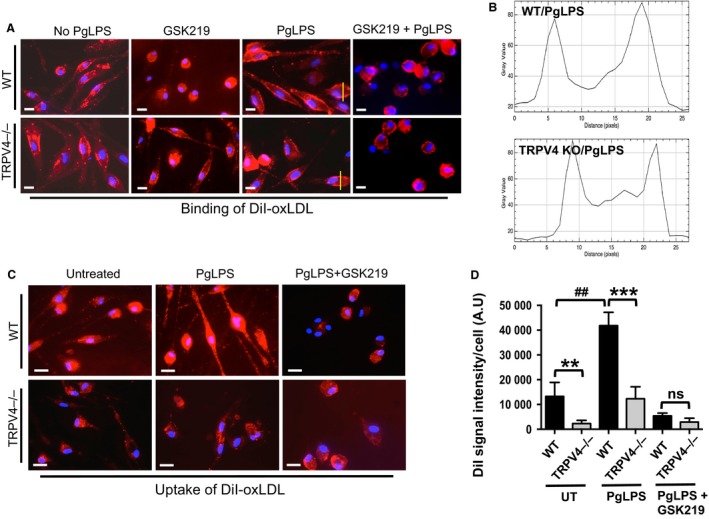
TRPV4 is required for oxLDL uptake but not its cell surface binding to macrophages. (A). WT and TRPV4 KO MRMs were incubated with or without DiI‐labeled oxLDL (2.5 *μ*g/mL) for 60 min at 4°C to assess oxLDL binding. Representative images of DiI‐oxLDL binding on the macrophage surface are shown (*n* = 5 fields/condition). (B) Quantification of results in Figure 6A using NIH ImageJ software. (C) WT and TRPV4 KO MRMs were incubated with or without DiI‐labeled oxLDL (5 *μ*g/mL) for 30 min at 37°C, and oxLDL uptake was assessed. Representative images from five different fields per condition are shown (original magnification, 40×). DiI‐oxLDL uptake indicated by red fluorescence. (D) Bar graphs show mean DiI fluorescence intensity (mean ± SEM) (NIH ImageJ software). **P < 0.01 for WT cells versus TRPV4−/− without PgLPS; ^##^ *P* < 0.01 for WT with PgLPS versus WT without PgLPS; ***P < 0.001 for WT with PgLPS versus TRPV4−/− with PgLPS;* n* = 20 cells/condition.

We analyzed binding and internalization of oxLDL in macrophages to investigate whether TRPV4 influenced macrophage foam cell formation in response to PgLPS by modulating these responses. WT and TRPV4 KO MRMs were incubated with fluorescent dye‐ labeled LDL (DiI‐oxLDL) at 4°C followed by 37°C (see Methods) to determine whether TRPV4 played a role in binding and uptake of oxLDL. Our results indicated similar binding of DiI‐oxLDL in TRPV4 KO cells compared to WT cells (Fig. 6A–B). Interestingly, TRPV4 KO MRMs exhibited significantly reduced DiI‐oxLDL uptake after 1 h compared with WT MRMs with or without PgLPS stimulation (Fig. 6C–D). Furthermore, we evaluated whether inhibition of TRPV4 channel activity in BMDMs by a selective small chemical inhibitor, GSK219, would influence internalization of DiI‐oxLDL. Our data showed that TRPV4 antagonism by GSK219 made no difference in oxLDL binding in WT MRMs stimulated with PgLPS (Fig. 6A). However, internalization of DiI‐oxLDL was significantly higher in untreated WT macrophages compared to GSK219‐treated cells after 1 h incubation (Fig. 6C–D). Taken together, these results suggest that TRPV4 activity regulates PgLPS‐induced oxLDL uptake but not it's binding at the macrophage surface.

## Discussion

The major findings of this study are: (1) TRPV4 is involved in oxLDL‐induced macrophage foam cell formation in response to *P. gingivalis* ‐derived lipopolysaccharide (PgLPS) or to increasing matrix stiffness, and (2) TRPV4‐elicited Ca^2+^ influx is augmented in response to PgLPS treatment. Mechanistically, we found that (1) TRPV4 regulates oxLDL internalization but not its cell surface binding in PgLPS‐treated macrophages, and (2) reduced foam cell generation in TRPV4 deficient cells was independent of expression of CD36, a scavenger receptor for oxLDL. Furthermore, we provide evidence that colocalization of TRPV4 and CD36 on plasma membrane is sensitive to changes in matrix stiffness in PgLPS‐treated cells.

It has been reported that exposure to *Porphyromonas gingivalis* lipopolysaccharide, an immunomodulatory molecule commonly found in the blood stream of patients with chronic periodontal disease, enhances binding and internalization of modified/oxidized LDL, induces macrophage foam cell formation, and aggravates M1 macrophage infiltration and macrophage‐mediated inflammation in infarcted tissue (Qi et al. 2003; Hayashi et al. 2011; Fukasawa et al. 2012; Teeuw et al. 2014; Chukkapalli et al. 2015; Schmitt et al. 2015; Houcken et al. 2016; Goswami et al. 2017). We have sought to determine the cellular and molecular mechanisms that regulate the binding and internalization of oxLDL within macrophages that may be responsible for generation of PgLPS‐induced foam cells. Our present data support the notion that TRPV4‐mediated Ca^2+^ influx integrates PgLPS‐induced signals to bolster macrophage foam cell generation. Considerable evidence suggests that oxLDL promotes Ca^2+^ influx and macrophage foam cell formation (Yang et al. 2000; Rahaman et al. 2011b). Interestingly, these oxLDL‐mediated effects were shown to be abrogated by nonspecific Ca^2+^ channel blockers (Yang et al. 2000; Rahaman et al. 2011b). Published reports from our laboratory and others have shown that TRPV4‐induced Ca^2+^ influx has diverse roles in different cell types including macrophages (Rahaman et al. 2014; Scheraga et al. 2016; Goswami et al. 2017; Sharma et al. 2017). Our current results show that TRPV4 augments oxLDL‐induced foam cell formation in response to PgLPS stimulation. We also showed that oxLDL‐induced expression of inflammatory cytokines was reduced in TRPV4 null cells. These results are consistent with our previous report that TRPV4 plays a role in oxLDL‐induced macrophage foam cell formation (Goswami et al. 2017).

Recent evidence documents an atheroprotective role of TRPV4 in which TRPV4 function in endothelial cells is linked to activation of eNOS and suppression of monocyte adhesion to endothelial cells (Xu et al. 2016). In contrast, impairment of TRPV4 channels has been linked to endothelial dysfunction, reduced macrophage foam cell generation, and vascular diseases (Zhang et al. 2009; Ye et al. 2012; Du et al. 2016; Goswami et al. 2017). In this study, we found that treatment with PgLPS results in upregulation of TRPV4 protein expression and TRPV4‐induced Ca^2+^ influx in macrophages. The kinetics of Ca^2+^ influx in BMDMs in response to TRPV4 agonist, GSK101, was much more gradual than the originally reported steep kinetics for GSK101 in HeLa cells transiently transfected with TRPV4 (Jin et al. 2011). It is possible that the differential kinetics pattern (steep vs. gradual) may be related to the origin of cells (overexpression of transfected TRPV4 in Hela cells vs. primary BMDMs). Recently, Scheraga et al. showed that TRPV4 activation is required for LPS‐induced macrophage phagocytosis and stimulation of inflammatory cytokines (Scheraga et al. 2016). We found that matrix stiffness altered PgLPS‐induced pro‐atherogenic responses such as oxLDL internalization and foam cell formation in a TRPV4‐dependent manner. In addition, we demonstrated that PgLPS exposure to macrophages on a stiff matrix induced increased TRPV4 Ca^2+^ influx activity. Collectively, our current results showed that TRPV4‐elicited Ca^2+^ influx integrates PgLPS‐ and matrix stiffness‐induced signals to mediate macrophage oxLDL uptake and foam cell formation. Furthermore, our data demonstrate that TRPV4 regulates the development of foam cells, possibly by regulating the internalization of oxLDL, but does not regulate the binding of oxLDL to the cell surface. Loss of TRPV4 function, either by genetic deletion or pharmacologic antagonism (by GSK219), abrogates PgLPS‐stimulated Ca^2+^ influx and uptake of oxLDL by macrophages growing on a stiff substrate.

Accumulating data support the notion that both a biochemical factor, for example, lipopolysaccharide, and a biomechanical factor, for example, matrix stiffness, may promote athero‐inflammatory macrophage function and atherosclerosis (Doherty et al. 1994; Shi et al. 1996; Yang et al. 2000; Melendez and Tay 2008; Nunes and Demaurex 2010; Moore and Tabas 2011; Rahaman et al. 2011a; Blakney et al. 2012; Kothapalli et al. 2012; Pi et al. 2014; Hind et al. 2015; Meng et al. 2015; Previtera and Sengupta 2015; Schmitt et al. 2015; Adlerz et al. 2016; Hansen and Taylor 2016; Houcken et al. 2016; Palombo and Kozakova 2016; Scheraga et al. 2016; Tedla et al. 2017). Since TRPV4 channels are sensitized by changes in biomechanical stimuli (Liedtke and Friedman 2003; Liedtke et al. 2003; Liedtke 2008; Adapala et al. 2013; Goswami et al. 2017; Sharma et al. 2017), we tested the hypothesis that TRPV4 modulates PgLPS‐induced proatherogenic macrophage functions in response to increased matrix stiffness. We found that accumulation of TRPV4 in plasma membrane in PgLPS‐stimulated macrophages was enriched by increases in matrix stiffness. Furthermore, we showed that PgLPS‐triggered enhancement of oxLDL‐induced foam cell generation was sensitive to changes in matrix stiffness. Altogether, these results suggest a possible mechanism by which function of TRPV4 proteins can be upregulated during PgLPS‐induced proatherogenic responses. Previously published reports have identified members of the TRP channel superfamily such as TRPC3 in macrophage survival, and have implicated TRPC3, TRPV2, and TRPM2 in macrophage phagocytosis, which might be of relevance to atherogenesis. Our results necessitate further studies to examine the role of TRPV4 on diverse functions of macrophages including migration, adhesion, apoptosis, and survival during atherogenesis. Efforts have been made to elucidate the mechanisms underlying foam cell formation with the goal of preventing atherosclerosis. Here, we identified a novel role of TRPV4 channels in PgLPS‐triggered exacerbation of macrophage foam cell formation, indicating an association of TRPV4 in proatherogenic processes in macrophages.

Our current data appear to have identified a specific plasma membrane receptor/channel, TRPV4, as a potential mediator of inflammatory/proatherogenic responses associated with pathogenesis of periodontitis‐induced atherosclerosis. Previous reports from our laboratory and others have shown a link between CD36‐mediated uptake of oxLDL and macrophage foam cell formation (Rahaman et al. 2006, 2011b; Moore and Tabas 2011; Moore et al. 2013). Since CD36 is the major scavenger receptor for oxLDL‐induced macrophage foam cell formation, we examined expression levels of CD36 in WT and TRPV4 KO cells. We found similar expression levels of CD36 protein in both WT and TRPV4 KO cells stimulated by PgLPS, suggesting that reduced foam cell formation in the absence of TRPV4 is not due to lack of CD36 expression. Thus, we postulate that augmented colocalization of TRPV4 and CD36 in response to increasing matrix stiffness in PgLPS‐treated macrophages may be linked to increased foam cell formation. A precise understanding of the mechanisms coupling periodontitis and atherosclerosis will be important to provide a rationale for long‐term longitudinal human studies required to assess causality, and to develop novel therapeutic interventions.

## Conflict of Interest

The authors declare that there is no conflict of interest.

## Data Availability Statement

The datasets generated during and/or analyzed in this study are available from the corresponding author on reasonable request.
